# The long-term outcome of trabeculotomy: comparison with filtering surgery in Japan

**DOI:** 10.1186/s12886-019-1107-0

**Published:** 2019-04-30

**Authors:** Wenjun Bao, Kazuhide Kawase, Hailong Huang, Akira Sawada, Tetsuya Yamamoto

**Affiliations:** Department of Ophthalmology, Gifu University Graduate School of Medicine, Gifu, 501-1194 Japan

**Keywords:** Trabeculotomy, Trabeculectomy, Intraocular pressure, Visual field

## Abstract

**Background:**

To investigate the long-term outcome of trabeculotomy and to compare it with that of trabeculectomy.

**Methods:**

We retrospectively reviewed the medical records of patients who had undergone standalone trabeculotomy. Inclusion criteria included a follow-up period of at least 6 years, availability of reliable static visual field results, etc. Age- and preoperative intraocular pressure -matched trabeculectomy cases served as controls. A Kaplan-Meier analysis was employed as a measure of surgical success. Additional clinical factors were also analyzed.

**Results:**

Twenty-five eyes of 25 trabeculotomy patients and 20 eyes of 20 trabeculectomy patients with a mean postoperative follow-up period of 8.0 years were selected. The Kaplan-Meier analysis estimated that the success probability defined as intraocular pressure < 16 mmHg was 44.0 ± 9.9% and 75.0 ± 9.7% at 6 years for trabeculotomy and trabeculectomy, respectively. The final mean deviation significantly progressed in trabeculotomy cases in Central 30–2 programs of the Humphrey Field Analyzer (*P* = 0.025). Patient characteristics and postoperative clinical data were analyzed by Mann-Whitney’s U test and Wilcoxon signed-rank test.

**Conclusions:**

While trabeculotomy was inferior to trabeculectomy in terms of intraocular pressure control and visual field stability in our series, surgical indications should always be determined on an individual basis, pending further research.

## Background

The most common surgical techniques for management of glaucoma are filtering surgery and outflow channel surgery focused on Schlemm’s canal. Trabeculotomy is an outflow channel surgery first reported by Burian and Smith in 1960. Harms subsequently modified the technique, employing a scleral flap to more easily identify Schlemm’s canal in 1970, which further popularized the surgery. There are many reports of good intraocular pressure (IOP) control via trabeculotomy in open- angle glaucoma, including childhood glaucoma, steroid-induced glaucoma, and exfoliation glaucoma [1–4]. The procedure is mainly indicated in younger patients, in eyes with higher IOP, or in eyes with milder glaucomatous changes. Trabeculotomy is characterized by incising the trabecular meshwork that is believed to play a role in the elevated intraocular pressure in many glaucoma subtypes. It is commonly performed with cataract surgery because it is more effective in this combined procedure.

Trabeculectomy was first reported by Cairns in 1968 [5]. After the introduction of adjunctive mitomycin C, it becomes the standard surgical procedure for most of the glaucoma subtypes [6, 7]. Although the ocular hypotensive effect of trabeculectomy is significant, several postoperative complications including bleb-associated endophthalmitis [8] hypotony maculopathy, etc. warn ubiquitous use of this surgical technique.

A variety of surgical procedures to reduce the resistance at the trabecular meshwork have been developed, which include several minimally invasive glaucoma surgeries (MIGSs) [9–12]. MIGS is an approach from the inside of the eye involving a small incision, and is notable for its minimally invasive nature and high biocompatibility. Because of its high safety, quick recovery, and the above-mentioned advantages, outflow channel surgeries have recently gained attention [13]. The common MIGS procedures involving the Schlemm’s canal include the removal of trabecular tissue (e.g.,Trabectome®: NeoMedix Corporation,CA,USA, Kahook®: New World Medical, CA,USA) [14, 15] or the implantation of a small device (e.g., iStent, iStent inject®, and Hydrus MicroShunt) [16, 17]. Since MIGS has only a short history, however, we do not know its long-term outcome. Because trabeculotomy is a classical type of outflow channel surgery and because it might serve as a surrogate, in a sense, for outflow channel surgery-type MIGS, it may be worthwhile to investigate the long-term prognosis of trabeculotomy and to compare it with that of standard trabeculectomy to predict the long-term IOP control of MIGS.

For above-stated reason, we evaluate and report the long-term results of trabeculotomy as compared with that of trabeculotomy in this study.

## Methods

### Study design

This is a retrospective, observational study. All methods were approved by the Ethics Committee of Gifu University Graduate School of Medicine, Gifu, Japan. The Ethics Committee approved the conduct of this research without obtaining written informed consent for this particular study under the general condition that any patient attending the Hospital has the right to refuse to provide his/her medical information to medical research.

### Patient selection and surgical procedures

We reviewed the medical records of all patients who had undergone standalone trabeculotomy at Gifu University Hospital between June 2004 and December 2010, and who fulfilled the following selection criteria: 1. primary open- angle glaucoma (POAG); 2. aged younger than 70 years old at the time of surgery; 3. no prior intraocular surgeries conducted; 4. followed at least 6 years; 5. best-corrected visual acuity of at least 20/25; 6. spherical equivalent of the refractive error greater than − 8.0D; 7. IOPs measured by Goldmann applanation tonometry; 8. perimetric examination conducted using a Humphrey Field Analyzer (HFA: Carl Zeiss Meditec, Dublin, CA, USA); 9. mean deviation (MD) of HFA Central 30–2 better than − 15.00 dB before surgery; and 10. preoperative and over 6-year postoperative fields available with good test reliability in both Central 30–2 (C30–2) programs. The definition of good test reliability was fixation loss < 20%, false positives < 33%, and false negatives < 33%. When both eyes were eligible, the first-operated eye was selected for the study. Perimetric examination with an HFA was conducted in principle every 6 months using the Swedish interactive thresholding algorithm (SITA) standard. Medication number [18] was defined as the total number of glaucoma eyedrops and oral carbonic anhydrase inhibitors. We assigned a 1 for each topical medication and a 2 for systemic carbonic anhydrase inhibitors. The basic indications for the surgery were as follows: Trabeculectomy was indicated for the cases with moderate to severe visual field loss or need of lower postoperative intraocular pressure, and trabeculotomy indicated for those with early stage of POAG. We selected only the patients with follow-up more than 6 years after trabeculotomy. Then matching with trabeculectomy cases was conducted in terms of age and follow-up period.

**Fig. 1:**
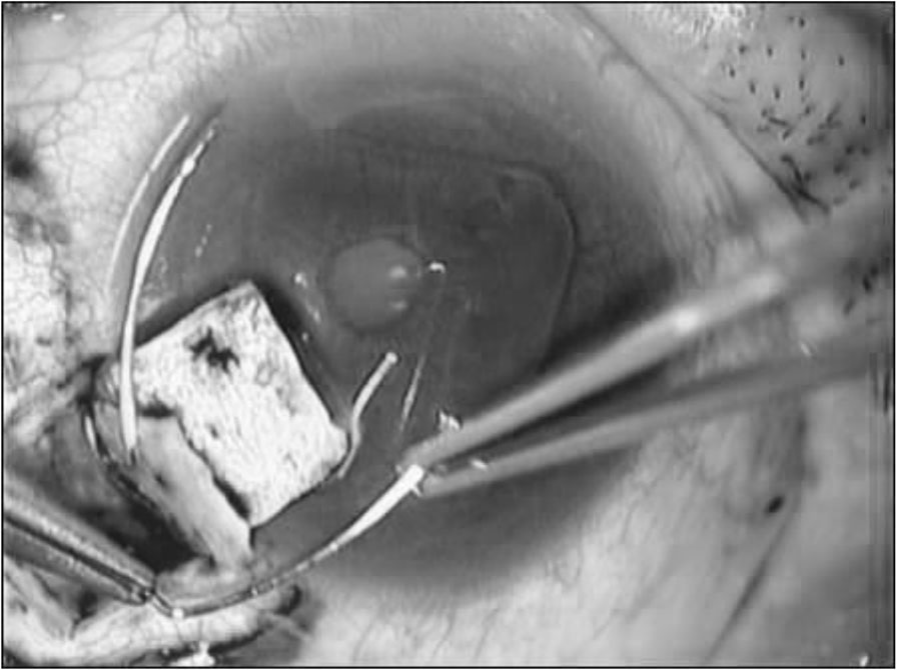
Intraoperative photograph of trabeculotomy

The trabeculotomy technique used in the present study was similar to that reported elsewhere [2], and proceeded as follows: 1. A limbal conjunctival incision was made. 2. A 4 × 4 mm square or triangular scleral double flap was created at the limbus. 3. After identification of Schlemm’s canal, its outer wall was cut with a razor blade and excised with fine scissors. 4. U-shaped probes (Nagata’s trabeculotome probe: Inami & Co., Ltd., Tokyo, Japan) were then inserted into both ends of the opened canal and rotated 90 degrees against the trabecular meshwork and toward the anterior chamber. 5. Rotation of these probes achieved a 120-degree opening of the trabecular meshwork (Fig. 1). 6. In some eyes, one or two sites of 1-mm-diameter sclerostomy through the scleral flap was performed with a punch, as suggested in a previous report [18]. 7. After removal of the inner flap, the scleral flap was closed with four 10–0 nylon sutures. 8. Conjunctival sutures were then placed.

We employed trabeculectomy cases operated adjunct with mitomycin C as controls for comparison with the trabeculotomy cases. Trabeculectomy cases that also fulfilled the selection criteria for trabeculotomy were matched with those undergoing trabeculotomy in age and preoperative IOP. The surgical techniques for trabeculectomy are reported elsewhere [19].

### Main outcome measure

The main outcome measure was the probability of success calculated by a Kaplan-Meier survival curve analysis. Surgical outcome was classified according to the postoperative IOP, and cases that had not reached to the following failure criteria were counted as a success. When an operated eye showed an IOP ≥ 21 mmHg (criterion A) or ≥ 16 mmHg (criterion B), either with or without ocular hypotensive medications, and that IOP level was recorded at two consecutive visits, the eye was rated a failure at the first visit during which that IOP was measured. IOP values measured within 3 months after surgery were not considered a surgical failure because the occurrence of postoperative IOP fluctuations after trabeculotomy is well known [20]. The eye was regarded as a surgical failure if additional glaucoma surgery was performed, or visual acuity deteriorated to an absence of light perception. We also compared postoperative clinical data including IOP, final medication number, and perimetric results.

### Statistical analysis

Data analysis was performed using the Bell Curve for Excel (Social Survey Research Information Co., Ltd., Tokyo Japan). The Kaplan-Meier survival curve analysis was conducted based on criterion A and criterion B, and the log-rank test was applied. Comparison of pre- and post-operative clinical data (preoperative IOP, postoperative mean IOP, final medication number) were performed using a Wilcoxon signed-rank test, and comparison between the trabeculotomy and trabeculectomy groups was performed using Mann-Whitney’s U test. The complications was analyzed by the Fisher exact test. Data was expressed graphically either as mean +/− 95% confidence interval or the mean overlaying individual data. A *p*-value of < 0.05 was considered to be statistically significant.

## Results

A total of 25 eyes of 25 patients and 20 eyes of 20 patients were selected in the trabeculotomy group and the matched trabeculectomy group, respectively. All were patients were Japanese. There was no significant difference in age, follow-up period, preoperative IOP, or medication score between the two groups. However, the mean deviation in the C30–2 measurements was significantly lower in the trabeculotomy group (Table 1).

**Table 1:** Patient characteristics

Preoperative data	Trabeculotomy	Trabeculectomy	P
Number of cases	25 eyes of 25 patients	20 eyes of 20 patients	–
Age (years)	41.8 ± 15.2 (18–69)	49.6 ± 9.8 (28–64)	0.120
Sex (Male/Female)	14/11	13/7	–
Glaucoma type	25	20	–
Mean follow-up period (years)	8.2 ± 1.9 (6.0–11.0)	7.8 ± 1.6 (6.0–11.0)	0.461
IOP (mmHg)	22.2 ± 6.8 (12.3–44)	24.0 ± 10.9 (13.7–56)	0.981
Medication number	4.4 ± 1.2 (2–7)	4.3 ± 1.4 (2–8)	0.563
HFA30–2 MD (dB)	−6.48 ± 4.07 (− 13.76 − + 0.27)	− 10.02 ± 3.63 (− 14.75 - -3.08)	0.009

*IOP* intraocular pressure, *MD* mean deviation, *HFA* Humphrey field analyzer

Mean ± SD (range), *P* Mann-Whitney’s U test

**Fig. 2:**
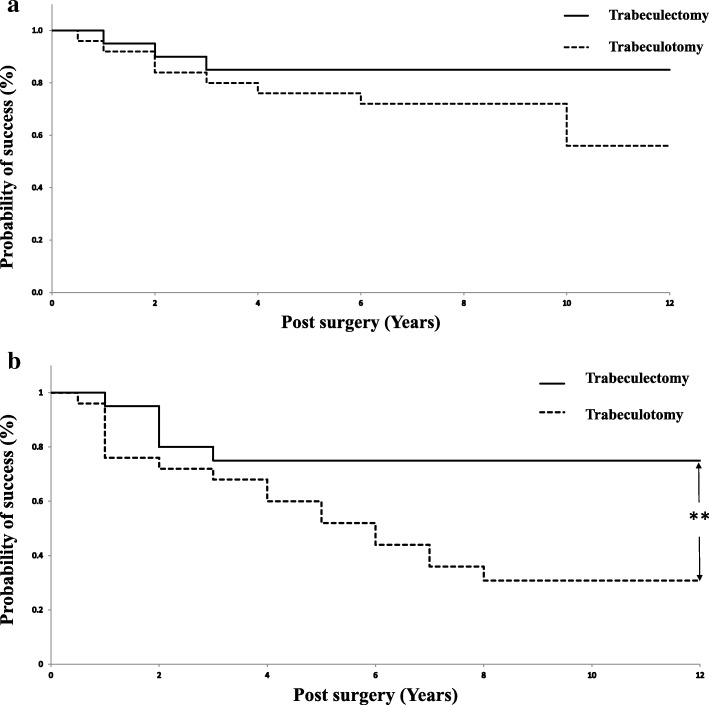
**a** Survival curve. Probability of success defined as IOP < 21 mmHg calculated with the Kaplan-Meier life-table analysis. Dotted line represents trabeculectomy, solid line represents trabeculotomy. No significant difference was found between the two groups (*P* = 0.170; log-rank test). **b** Survival curve. Probability of success defined as IOP < 16 mmHg calculated with the Kaplan-Meier life-table analysis. Dotted line represents trabeculectomy, solid line represents trabeculotomy. There was significant difference between the two groups (*P* = 0.012; **: *P* <  0.05; log-rank test)

The Kaplan-Meier analysis based on criterion A (IOP < 21 mmHg) estimated that the probability of successful IOP control (cumulative probability ± standard error) was 72.0 ± 9.0% and 85.0 ± 8.0% at 6 years (mean ± 95% CI *n* = 19 and 17) for the trabeculotomy group and the trabeculectomy group, respectively (Fig. 2a). There was no significant difference between the two groups (*p* = 0.170; log-rank test). By contrast, the criterion B success (IOP < 16 mmHg) according to the Kaplan-Meier analysis showed a probability of successful IOP control of 44.0 ± 9.9% and 75.0 ± 9.7% (mean ± 95% CI *n* = 13 and 15) at 6 years for the trabeculotomy group and the trabeculectomy group, respectively (Fig. 2b). The trabeculectomy group showed significantly higher probability of success than did the trabeculotomy group (*p* = 0.012; log-rank test).

**Fig. 3:**
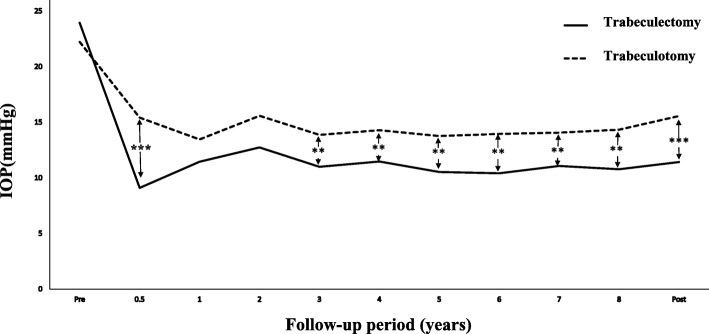
Course of IOP. Postoperative IOP was significantly lower in the trabeculectomy group compared with the trabeculotomy group, except at 1 and 2 years post-surgery (**: P <  0.05, ***: *P* <  0.001; Mann-Whitney’s U test)

**Fig. 4:**
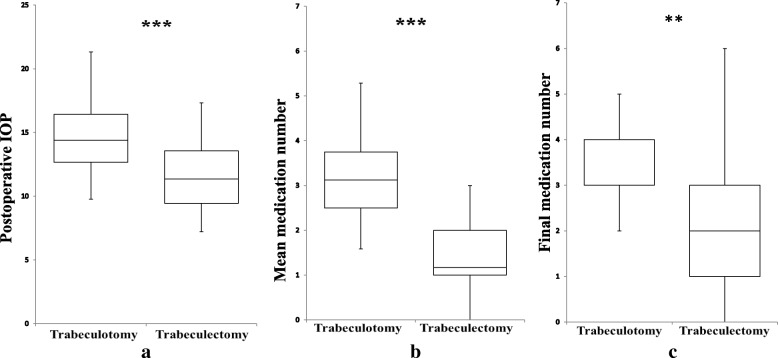
**a-c** Clinical data. The mean postoperative intraocular pressure (a), mean medication number (b) and final medication number (c) were significantly lower in the trabeculectomy group compared with the trabeculotomy group(**: *P* <  0.05,***: *P* < 0.001; Mann-Whitney’s U test)

Figure 3 shows that IOP significantly decreased during the entire follow-up period in both treatment groups (Wilcoxon signed rank test). The mean IOP, final IOP, and mean medication number were significantly lower in both groups as compared with the preoperative period (Fig. 4a-c). Trabeculotomy was associated with a significantly higher final medication score than was trabeculectomy. The final postoperative MD progressed significantly in the trabeculotomy group in C30–2 (*P* = 0.025; Wilcoxon signed rank test), but there was no significant progression in the trabeculotomy group (*P* = 0.156; Wilcoxon signed rank test) (Table 2).

**Table 2:** Postoperative clinical data

Postoperative data	Trabeculotomy	P*	Trabeculectomy	P*	P^#^
Mean IOP (mmHg)	15.6 ± 4.5 (9.8–29.0)	<0.001	11.4 ± 2.7 (7.2–17.3)	<0.001	< 0.001
Final IOP (mmHg)	14.8 ± 4.0 (9–25)	0.001	10.8 ± 2.5 (6–15)	<0.001	0.003
Final medication number	3.6 ± 1.8 (0–7)	0.108	2.2 ± 2.0 (0–7)	0.002	0.004
Mean medication number	3.3 ± 1.5 (0.2–7)	0.004	1.6 ± 1.1 (0–4.6)	<0.001	< 0.001
Final MD of HFA30–2 (dB)	− 7.90 ± 5.36 (− 17.73 − + 1.53)	0.025	−11.43 ± 5.21 (− 21.32 - -2.22)	0.156	0.136

Mean ± SD (range)

*: Wilcoxon signed-rank test of pre- and postoperative data

#: Mann-Whitney’s U test: comparison between trabeculotomy and trabeculectomy

Table 3 shows postoperative complications. Hyphema occurred in 68 and 30% following trabeculotomy and trabeculectomy, respectively. It occurred more frequently in trabeculotomy group (*p* = 0.017; Fisher exact test). Some eyes suffered from two or more complications.

**Table 3:** Postoperative complications

	Trabeculotomy (*n* = 25)	Trabeculectomy (*n* = 20)	P-value
Early complications			
Hyphema	17 (68%)	6 (30%)	0.017
Shallow anterior chamber	4 (16%)	5 (25%)	0.482
Choroidal detachment	0	2 (10%)	0.192
Hypotony maculopathy	0	2 (10%)	0.192
Late complications			
Cataract	1 (4%)	3 (15%)	0.309
Endophthalmitis	0	1 (5%)	0.444
Late onset Leaking bleb	0	1 (5%)	0.444

P-value: Fisher exact test

## Discussion

We investigated the efficacy of trabeculotomy in cases followed for at least 6 years (mean: 8.2 years), and found it to be inferior to trabeculectomy in terms of IOP control and visual field stability. Since MIGS has gained increasing attention among glaucoma specialists, the present study provides important insights which suggest that trabeculotomy, and possibly outflow channel surgeries in general, have some intrinsic limitations that should be considered in the surgical management of glaucoma.

Rosenquist et al. reported that enucleated human eyes irrigated at 25 mmHg showed a 30% reduction in total aqueous outflow resistance when 30^o^ of the trabecular meshwork was treated by trabeculotomy, and similarly 60% by 120^o^ trabeculotomy and 71% by 360^o^ (complete trabeculotomy) [21]. Trabeculotomy is already an established surgical technique, and the majority of procedures employ a 120-degree trabecular meshwork incision. Thus, we can expect some 60% reduction of trabecular meshwork resistance as a baseline. In the present study, the mean IOP was 22.1 mmHg preoperatively and decreased to 15.4 mmHg postoperatively. This reduction is consistent with the findings of Rosenquist et al. In a large trabeculotomy study, Tanihara et al. reported that the mean IOP ranged from 15.7 to 16.9 mmHg in eyes rated as success with or without medication at the end of a follow-up period that was similar to that used in the present study. As for the rate of IOP controlled less than 20 mmHg, Nambu et al. reported 62.2% in 8 years [22], whereas it is 72.0% in 6 years in the present study; The IOP control rate is similar. In contrast, it was reported for trabeculectomy to be 74.1% in 8 years [23], which is again similar to 85.0% in 6 years in the present study. How about the change in visual function following the two surgical techniques? Visual field change is the central concern in the management of glaucoma. Trabeculotomy was less effective in maintaining the visual field as compared with trabeculectomy in the present study.

Trabeculotomy is associated with several significant complications affecting visual prognosis, such as Descemet’s membrane detachment and transient, but significant, IOP elevation. However, the incidence, of such complications is relatively low [2, 18]. The rate of postoperative complications found in the present study was almost identical to the previous study. We confirmed in the present study that trabeculotomy is superior to trabeculectomy in terms of postoperative complications. On the contrary, trabeculectomy may also cause several sight-threatening complications including bleb-associated endophthalmitis and hypotony maculopathy, and the incidence of these complications is somewhat higher [8, 24–27] than that for the other complications. In the present study, however, these was only one case of hypotony maculopathy, thus rendering trabeculotomy superior to trabeculectomy in terms of postoperative complications within the present study [28–32].

Given that trabeculotomy is inferior in terms of both IOP control and visual field prognosis, albeit with lower rates, its indication must remain limited. Therefore, we recommend against the ubiquitous application of trabeculotomy in cases of open angle glaucoma requiring surgical intervention.

We must also consider the relevance of the present findings for understanding the new generation of outflow channel surgeries. Since many of these surgeries have only been recently developed, the lack of long-term outcome data prevents clear recommendations. In cases employing iStent™ and Hydrus™ devices [33–36], the length of the opened trabecular meshwork is less than that in trabeculotomy, so the ocular hypotensive effect is unlikely to represent an improvement. Perhaps the application of a more aggressive technology, such as those comprising a 360^o^ trabeculotomy or Gonioscopy-assisted Transluminal Trabeculotomy [37–40], can improve the hypotensive efficacy. In any case, the expected efficacy may be less than that seen in the trabeculotomy experiments reported by Rosenquist et al. [18]. As for MIGS, Trabectome was approved in 2010 in Japan. The success rate of Trabectome standalone surgery was reported some 50% in 2 years defining the success as the postoperative IOP of less than 21 mmHg and percent IOP reduction at least 20% [41]. The success rate seems to be better in our study even though the definition of IOP control is different. Outflow channel surgery has fewer complications as compared to trabeculectomy. Its ocular hypotensive effect, however, was inferior to trabeculectomy in a long run. These issues should be also considered in MIGS. Careful selection of an appropriate surgical technique is definitely needed for better management of each individual patient.

There are several limitations in the present study. First, it is a retrospective study. Second, the number of subjects is relatively small. It is because we enrolled trabeculotomy cases with a long-term follow-up and matched trabeculectomy cases were selected. Third, the indications for both surgical techniques were different. It may have affected postoperative IOP and survival rate. Therefore, care should be taken in applying the approaches described here to specific cases. However, the present study provides some empirical support for decisions regarding the proper indications for trabeculotomy.

## Conclusions

We investigated the long-term outcome of trabeculotomy compared with trabeculectomy and found the former provided inferior IOP control and visual field stability. These findings provide further empirical support for surgical decision making, although further research is required to more precisely identify the factors determining indications for any specific patient.

## Abbreviations

HFA: Humphrey Field Analyzer
IOP: Intraocular pressure
MD: Mean deviation
MIGSs: Minimally invasive glaucoma surgeries
POAG: Primary open-angle glaucoma
SITA: Swedish interactive thresholding algorithm
